# Human free-operant performance varies with a concurrent task: Probability learning without a task, and schedule-consistent with a task

**DOI:** 10.3758/s13420-019-00398-1

**Published:** 2020-01-02

**Authors:** Phil Reed

**Affiliations:** Department of Psychology, Swansea University, Singleton Park, Swansea, SA2 8PP UK

**Keywords:** Schedules of reinforcement, Response rate, Bout-initiation, Within-bout responding, Probability learning, Humans

## Abstract

Three experiments examined human rates and patterns of responding during exposure to various schedules of reinforcement with or without a concurrent task. In the presence of the concurrent task, performances were similar to those typically noted for nonhumans. Overall response rates were higher on medium-sized ratio schedules than on smaller or larger ratio schedules (Experiment 1), on interval schedules with shorter than longer values (Experiment 2), and on ratio compared with interval schedules with the same rate of reinforcement (Experiment 3). Moreover, bout-initiation responses were more susceptible to influence by rates of reinforcement than were within-bout responses across all experiments. In contrast, in the absence of a concurrent task, human schedule performance did not always display characteristics of nonhuman performance, but tended to be related to the relationship between rates of responding and reinforcement (feedback function), irrespective of the schedule of reinforcement employed. This was also true of within-bout responding, but not bout-initiations, which were not affected by the presence of a concurrent task. These data suggest the existence of two strategies for human responding on free-operant schedules, relatively mechanistic ones that apply to bout-initiation, and relatively explicit ones, that tend to apply to within-bout responding, and dominate human performance when other demands are not made on resources.

Exposure to schedules of reinforcement in nonhumans produces consistent and reliable patterns of behavior (Ferster & Skinner, 1957; Zeiler, 1984) that have formed the basis of theoretical accounts of the processes underling free-operant performance (Baum, 1981; Morse, 1966; McDowell & Wixted, 1986; Peele, Casey, & Silberberg, 1984; Tanno & Silberberg, 2012). However, human performance when exposed to these contingencies is more variable, suggesting additional factors influence their free-operant behaviors (Lowe, 1979; Pérez et al., 2016). In the same way that many species differences in learning can be attributed to procedural factors in experiments (Smith, McLean, Shull, Hughes, & Pitts, 2014), procedural factors may be responsible for human/nonhuman schedule differences, such as the need to involve response costs (Bradshaw & Reed, 2012; Matthews, Shimoff, Catania, & Sagvolden, 1977), consummatory responses (Pérez et al., 2016; Raia, Shillingford, Miller, & Baier, 2000), and meaningful outcomes as reinforcers (Lowe, Harzem, & Bagshaw, 1978; Reed, 2001). Other factors related to human/nonhuman differences involve personality traits and psychopathologies, such as depression (Alloy & Abramson, 1979; Dack, McHugh, & Reed, 2009) and schizotypy (Randell, Ranjith-Kumar, Gupta, & Reed, 2009).

The role of language in human schedule performance has been highlighted by many as important (Bradshaw & Reed, 2012; Hayes, Brownstein, Zettle, Rosenfarb, & Korn, 1986; Leander, Lippman, & Meyer, 1968; Lowe, 1979; Shimoff, Matthews, & Catania, 1986). Human free-operant performance can be related to participant verbalizations about the nature of the schedule (Bradshaw & Reed, 2012; Leander et al., 1968; Matthews et al., 1977). There are instances in which accurate verbalization of the nature of the relationship between performance and the received outcomes—performance awareness (Bradshaw, Freegard, & Reed, 2015; Hayes et al., 1986)—is associated with human free-operant responding being similar to that seen for nonhumans (Bradshaw & Reed, 2012). It has been shown that verbal rules generated about the schedule (Bradshaw et al., 2015; Hayes et al., 1986), or instructions given to humans prior to a free-operant task (Matthews et al., 1977; Shimoff et al., 1986), impact strongly on their performance, often to a greater extent than the actual contingency (Catania, Matthews, & Shimoff, 1982; Hayes et al., 1986). However, there are instances where humans show accurate performance awareness, but do not show typical schedule performance, often due to interactions between self-generated rules, experimenter-provided rules, and response outcomes (Fox & Kyonka, 2017; Hackenberg & Joker, 1994).

Reed (2015a; Reed, Smale, Owens, & Freegard, 2018) employed a concurrent task (counting backwards out loud) designed to reduce the potential impact of verbalizations on human schedule performance, and noted that, with the task, human schedule behavior was highly similar to that seen in nonhumans: responding was faster on random ratio (RR) than on random interval (RI) schedules, rates of response were associated with interval and ratio values as they are for nonhumans. In fact, the impact of a concurrent load on human schedule performance has theoretical relevance. Concurrent tasks not only interfere with language (Andersson, Hagman, Talianzadeh, Svedberg, & Larsen, 2002) but also reduce levels of explicit awareness and/or processing resources that participants can devote to a task (Fu & Anderson, 2008), and reduce the degree to which attention can be paid to multiple aspects of contingencies (Green & Flowers, 2003; Mitchell, De Houwer, & Lovibond, 2009; Reynolds & Reed, 2011). Aspects of free-operant contingencies that require explicit awareness, or greater processing resources, such as the ability to track the relationship between the occurrence of outcomes and actions (Mitchell et al., 2009; Newell et al., 2007), have been given prominence in the investigation of human free-operant performance (Dickinson & Perez, 2018; Pérez et al., 2016; Reber, 1989).

There are a number of ways of characterizing such ‘molar’ aspects of the contingency, but one that seems important is the correlation between response rate and reinforcement rate (Dickinson & Perez, 2018) or the response–reinforcer feedback function (McDowell & Wixted, 1986; Reed, 2015b). Pérez et al. (2016) suggest that ability to track action–outcome relationships may be responsible for driving goal-directed responding by humans on free-operant schedules (see also Reed et al., 2018), and that this aspect of the contingency will drive performance more strongly on ratio than on interval schedules, where this action–outcome relationship is stronger (see also Baum, 1993; McDowell & Wixted, 1988). In contexts other than free-operant learning, the ability to engage in such strategies that require integration of information over time is impaired by the presence of a demanding concurrent task (Cohen, Ivry, & Keele, 1990; Green & Flowers, 2003). Thus, reduced cognitive resources occasioned by the presence of a concurrent task may impede human’s ability to attend to such aspects of the contingency.

An inability to engage in such explicit or higher-level processing might impact the manner in which free-operant contingencies affect behaviour, and different schedule performance by humans might be noted with and without concurrent loads. When these abilities/resources are available, human free-operant performance could relate strongly to such action–outcome relationships (i.e., the probability of a reinforce given a response, or the response–reinforcer feedback function); but, in the absence of these abilities/resources, human performance could be influenced by ‘mechanistic’ factors, more typically operative in nonhumans (but not always so; Reed, 2015b). These mechanistic/implicit processes may be overridden when participants have greater available cognitive resources to attend to other dimensions of the task (such as the response–reinforcer rate relationship) on which explicit processes could operate (Hayes et al., 1986; Pérez et al., 2016; Reed, 2001; Song, Howard, & Howard, 2007). However, the impact of concurrent loads on human schedule-performance has not been explored, which is the primary aim of the current series of experiments.

For example, rate of reinforcement drives some aspects of responding on free-operant schedules (the tendency to commence sequences of responding—bout-initiation responding), possibly through conditioning the context, which serves to drive ongoing responding (Reed et al., 2018). Additionally, other aspects of responding (the tendency to continue responding once initiated—within-bout responding) are controlled by the reinforcement of different interresponse times (IRTs; Tanno & Silberberg, 2012).

In addition to this primary aim, exploration of the impacts of a concurrent load on the microstructure of free-operant responding may be helpful in understanding the nature of human instrumental responding. Many have suggested that instrumental learning comprises at least two mechanisms: a stimulus–response (S–R) mechanism that drives responding as a function of the previous relationship between the context and reinforcement, and goal-directed responding (e.g., Balleine & Dickinson, 1998; Dolan & Dayan, 2013; Pérez et al., 2016). The latter form of responding is taken to be more under the control of explicit process and might be expected to be affected to a greater extent by the presence of a concurrent load, but this is currently unknown.

A range of procedures assessing the microstructure of schedule behavior have shown free-operant performance is composed of two distinct types of responses: bout-initiation responses, which instigate a bout of responding, and within-bout responses, which follow the initial response and compose the response bout (Bowers, Hill, & Palya, 2008; Killeen, Hall, Reilly, & Kettle, 2002; Mellgren & Elsmore, 1991; Pear & Rector, 1979; Reed, 2011, 2015a; Shull, 2011; Shull, Gaynor, & Grimes, 2001). These two forms of free-operant response are differentially sensitive to aspects of the contingency: bout-initiation responses are controlled by the rate at which reinforcement has been delivered in the conditioning context (Reed, 2011, 2015a; Shull, 2011; Shull et al., 2001), and, thus, potentially by contextual conditioning (Reed et al., 2018). Speculatively, it may be that these are ‘mechanistic S–R responses’, which may not be impacted by concurrent loads, as they require less explicit processing (see Balleine & Dickinson, 1998). In contrast, within-bout responses are not related to this factor to such a degree (Brackney, Cheung, Neisewander, & Sanabria, 2011; Killeen et al., 2002; Reed, 2011; Shull et al., 2001). These responses may be related to either the response–reinforcer feedback function (perhaps an explicit process requiring greater cognitive resources), or the role of reinforcement in shaping interresponse times (perhaps a more mechanistic process requiring fewer resources). However, it is currently unknown if these within-bout responses, due to their dependence on their relationship to the goal, may be more affected by procedures reducing explicit processing. A secondary aim of the current studies is to explore whether any evidence can be noted that this distinction may be obtained using the current procedures.

To explore the microstructure of responding, two different procedures were adopted to ensure that the qualitative pattern of results obtained was not dependent upon any particular procedures or assumptions regarding the data. Bout-initiation and within-bout responses have been studied using log survival plots of IRTs (Killeen et al., 2002; Shull et al., 2001). A frequency distribution for emitted IRTs is created, and the percentage IRTs emitted in a particular time bin calculated as a proportion of all IRTs not yet emitted (i.e., those that fall into that and the later time bins). These survivor percentages are turned into logs, and a ‘log survivor plot’ is generated; Shull et al., 2001). In a log survivor plot, the slope between any two points is an indicator of the relative decline in the proportion of the IRTs per opportunity between those points and indicates response rate: The steeper the slope, the higher the relative rate of responding during the interval. Shull et al. (2001; see also Brackney et al., 2011; Killeen et al., 2002; Sibley, Nott, *&* Fletcher, 1990) found that the slope of log survival plots for rats performing on free-operant schedules of reinforcement was not uniform, but rather comprised an initially negative slope, followed by a portion with a shallow negative slope. This pattern of data was interpreted as indicating the presence of two different types of responding: a set of shorter IRTs prior to the break point (i.e., the point at which the slope of the line changes) reflecting ‘within-bout’ responding; and a set of longer IRTs following the ‘break’ point classed as ‘bout-initiation’ responses. A double exponential equation can be fitted to these data, where the equation fits the two distributions of IRTs (i.e. those prior to the ‘break’ taken to represent response initiations; and those after the break, taken to represent within-bout responses. This equation takes the form: Ppred = a × exp(−bt) + (1 − a) × e(−dt), where b and d represent the rates of within-bout and bout-initiation, respectively.

Although this approach has been used for humans (see Reed et al., 2018), it requires certain assumptions to be made about the distribution of the data in order to fit the double exponential equation, which may or may not be present in the human case. In contrast, the approach suggested by Mellgren and Elsmore (1991; Reed, 2011, 2015a; Sibley et al., 1990) designates responses following a short interresponse time (IRT) as within-bout responses, and those following a long IRT as bout-initiation responses. This approach is conceptually simple, and does not suffer from problems of making assumptions about the distribution of responding required for an equation-fitting approach (Bowers et al., 2008; Shull, 2011), or altering the nature of the contingency away from a typically studied schedule (Reed, 2011). However, it does make assumptions about which responses should be regarded as bout-initiating and within-bout. Thus, both approaches have different shortcomings, but if both show similar patterns of relationships between the two types of responding and schedule factors, then this will give greater confidence that the results are not the product of a procedural artefact.

Given the above, when explicit processes are made less available, due to the presence of a cognitive load, human schedule performance might resemble that seen in nonhumans. Moreover, there might be differential effects of a concurrent load on the two forms of free-operant responses, with within-bout responding being more sensitive to the impacts of a concurrent load than bout-initiation responding.

## Experiment 1

The first experiment explored the impact of a concurrent task on human performance on random ratio (RR) schedules. On RR schedules, nonhumans emit response rates that have an inverted-U relationship to ratio size, at least over a range of ratio values up to RR-60. That is, response rates tend to be low for small ratio values, high for medium ratio values, and decrease again for high ratio values (Ferster & Skinner, 1957; Reed & Hall, 1988). This pattern of data would not be expected based on a view that suggests responding is driven by aspects of the relationship between responding and reinforcement over time (either probability of an outcome given a response, Chatlosh, Neunaber, & Wasserman, 1985; or the response–reinforcer feedback function, McDowell & Wixted, 1988; Pérez et al., 2016). If these aspects of the contingency require greater cognitive resources, and this form of processing is made harder by the presence of a concurrent task, then an inverted-U function should be seen in the presence of a load, but not in its absence (cf. Pérez et al., 2016; Reed, 2015a).

In terms of the two forms of schedule responses, the above U-function pattern of responding is seen for within-bout responding in nonhumans (Reed, 2011), and for humans with a concurrent load (Reed, 2015a). However, in the absence of such a load, if the relationship between responding and reinforcement were responsible for human schedule performance, and responding were related to the chances of receiving an outcome for a response, then with-bout response rates should decrease as the ratio value increases (Chatlosh et al., 1985; Pérez et al., 2016). This prediction would hold irrespective of whether a simple outcome-given-a-response probability (Chatlosh et al., 1985), or a response–reinforcer feedback function (McDowell & Wixted, 1988), view of this aspect of the contingency were taken. In contrast, bout-initiation responses tend to be related to the rate at which reinforcement is delivered irrespective of the nature of the schedule (Killeen et al., 2002; Reed, 2011). It may be that such responding already reflects a more mechanistic, or less cognitively demanding process, and would not be affected by the presence of a cognitive load.

### Method

#### Participants

Forty-five participants (25 female and 20 male), with a mean age of 20.15 (±2.44, range: 18–26) years, were recruited. The participants were students at Swansea University and received Psychology Department subject-pool credits for their time and the possibility of a prize at the end of the study. All participants had normal or corrected-to-normal vision and were naïve to the experiment’s purpose. As previous studies have demonstrated that individuals scoring highly in terms of depression and schizotypy show atypical patterns of schedule performance (Dack et al., 2009; Randell et al., 2009), participants with high psychometric test scores in these areas were excluded; five participants were excluded on this basis, leaving 40 participants in the study.

#### Apparatus

The experimental task was presented using Visual Basic (6.0) on a laptop computer with a 40-cm screen. The program presented an RR schedule (10, 30, or 60) to the participants. On a particular schedule, each response (a space-bar press) had an equal probability of reinforcement (i.e., 1/10, 1/30, or 1/60). Each participant began the experiment with 40 points, displayed in a box, under the word *points*, in the middle of the screen, approximately one third of the way from the bottom of the screen. An 8-cm wide × 3-cm high colored square (either blue, purple, or yellow) was displayed in the middle of the screen, approximately one third from the top of the screen, to differentiate the different conditions. Reinforcement consisted of 60 points being added to the ‘points’ box. Each response subtracted one point from the ‘points’ box, which aimed to prevent a lack of performance regulation in humans that can occur when there is no cost for a response (Bradshaw & Reed, 2012; Reed, 2015a; Reed et al., 2018).

#### Measures and tasks

The Oxford–Liverpool Inventory of Feelings and Experiences–Brief Version (O-LIFE[B]; Mason, Linney, & Claridge, 2005) measures schizotypy (Cronbach α = .62 to .80). A score of greater than 6 on the Unusual Experiences scale (one standard deviation above the mean; Mason et al., 2005) was taken as a cutoff for individuals displaying high levels of this trait, who display atypical performance (Randell et al., 2009).

Beck’s Depression Inventory (BDI; Beck, Ward, Mendelson, Mock, & Erbaugh, 1961) assesses depression (Cronbach α = .73 to .92; Beck, Steer, & Garbin, 1988). A score higher than 10 was taken as a cutoff for individuals displaying high levels of depression, producing atypical schedule performance (Dack et al., 2009).

Concurrent task (Andersson et al., 2002) participants counted backwards, out loud, in 7 s from a random five-digit number. To enhance task adherence, a recording device was placed prominently on the desk in front of the participant, and they were told that their answers to the counting task would be analyzed and scored later. Participants were prompted to continue the counting task if they paused.

#### Procedure

Participants were tested individually in a quiet room containing a desk, a chair, and a computer. The following instructions were presented on the computer screen: *You have to score as many points as possible by pressing the space bar on the computer. The coloured shape may be important. To receive points, sometimes you might need to press the space bar quickly, and at other times you might need to press slowly. The person with the best score will receive a £50 [name of company] token*. In addition, participants in the concurrent task group (but not the control group) were also told: *You must count backwards, out loud, in 7 s, from the number XX,XXX.*

Each participant was then exposed to all three schedules (RR-10, RR-30, and RR-60). Each schedule was presented once to each participant for 10 min, with a 30-s intercomponent interval. Each different schedule was signaled by a different colored rectangle on the screen. The particular colors used to signal the schedules, and the order of schedule presentation, were randomized. Each response subtracted 1 point from the ‘points’ box displayed on the screen. Reinforcement consisted of the addition of 60 points to the ‘points’ box. Half of the participants (*n* = 10) were assigned to the control group, and these were the only contingencies in operation. The other participants (*n* = 10) were assigned to the concurrent task group, and had to perform the counting backwards task as they were performing the schedule task. Following the experimental task, participants completed both the BDI and the O-LIFE(B) scales.

### Results and discussion

**Fig. 1:**
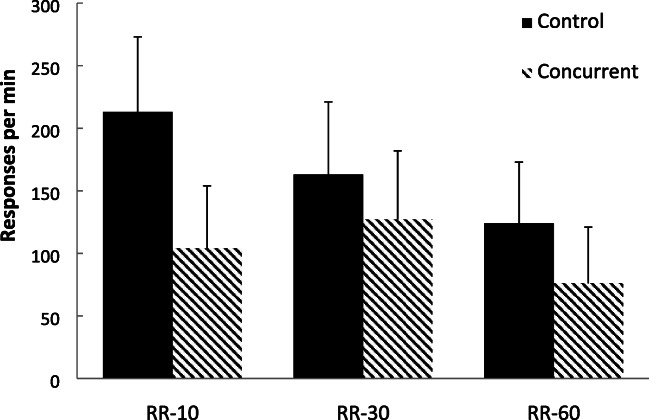
Experiment 1. Group-mean response rates for the three schedules. Control = no concurrent task. Concurrent = concurrent counting backwards task. Error bars = 95% confidence intervals

Figure 1 displays the group-mean rates of responding across the three schedule types over the last 5-min exposure to each schedule to reduce any carryover effects (Reed, 2015a). For the control group (lacking a concurrent task), response rates decreased as the ratio value increased. However, for the concurrent task group, response rates were highest for the RR-30 condition, compared with the RR-10 and RR-60 schedule conditions A two-factor mixed-model analysis of variance (ANOVA), with group (control versus concurrent) as a between-subject factor, and schedule (RR-10, RR-30, RR-60) as a within-subject factor, was conducted on these data. This analysis revealed that there was only a marginally significant main effect of group, *F*(1, 38) = 3.85, *p* = .057, η_p_^2^ = .092, 95% CI [.000, .282], *p(H*_*1*_*/D)* = .522, a significant main effect of schedule, *F*(2, 76) = 18.71, *p* < .001, η_p_^2^ = .330, [.155, .461], *p(H*_*1*_*/D)* = .987, and a significant interaction between the two factors, *F*(2, 76) = 7.71, *p* < .001, η_p_^2^ = .169, [.034, .308], *p(H*_*1*_*/D)* = .502. Polynomial contrast analyses conducted for each group revealed a significant linear trend, *F*(1, 19) = 39.25, *p* < .001, η_p_^2^ = .674, [.360, .794], *p(H*_*1*_*/D)* = .999, but not a quadratic trend, *F* < 1, η_p_^2^ = .008, [.000, .202], *p(H*_*o*_*/D)* = .502, for the control group. This means that response rates decreased as the ratio value increased for the control group. In contrast, there was no significant linear trend, *F*(1, 19) = 2.68, *p* = .118, η_p_^2^ = .124, [.000, .387], *p(H*_*o*_*/D)* = .543, but a significant quadratic trend, *F*(1, 19) = 22.63, *p* < .001, η_p_^2^ = .544, [.194, .712], *p(H*_*1*_*/D)* = .999 for the concurrent task group. This means that response rates increased, and then decreased, as the ratio value increased for the concurrent group.

These data corroborate previous investigations of human RR schedule performance with a concurrent task (Reed, 2015a), in that response rates followed the pattern sometimes observed for nonhumans; responding showed an inverted-U function over the current set of ratio parameters (Ferster & Skinner, 1957; Reed & Hall, 1988). In contrast, when a concurrent task was not employed, response rates on the schedules decreased as the ratio value increased. This effect has been noted on contingencies that have been designed to study probability effects (Chatlosh et al., 1985; Pérez et al., 2016). This pattern of results is consistent with the view that when cognitive resources are available, mechanisms operating on the integration of information over time, such as those relating the rates of responding and reinforcement, may be more dominant than when these relationships are available to a lesser extent. Under these latter conditions, human free-operant responding resembles that of nonhumans, and may be driven by more mechanistic processes.

The equation: Ppred = a × exp(−bt) + (1 − a) × e(−dt), where b and d represent the rates of within-bout and bout-initiation, respectively, was fitted for each participant individually, by entering the individual’s IRTs into the spreadsheet developed by Peter Killeen (available on the SQAB website, and later modified by Richard Shull). The worksheet fits the data by minimizing the summed squared differences between the logs of obtained and predicted survivor proportions. It also excludes the longest 1% of IRTs, as very long IRTs may result from extra-experimental factors, thus the programme forces a better fit to the right tail—the portion relevant to bout-initiation rate.

**Fig. 2:**
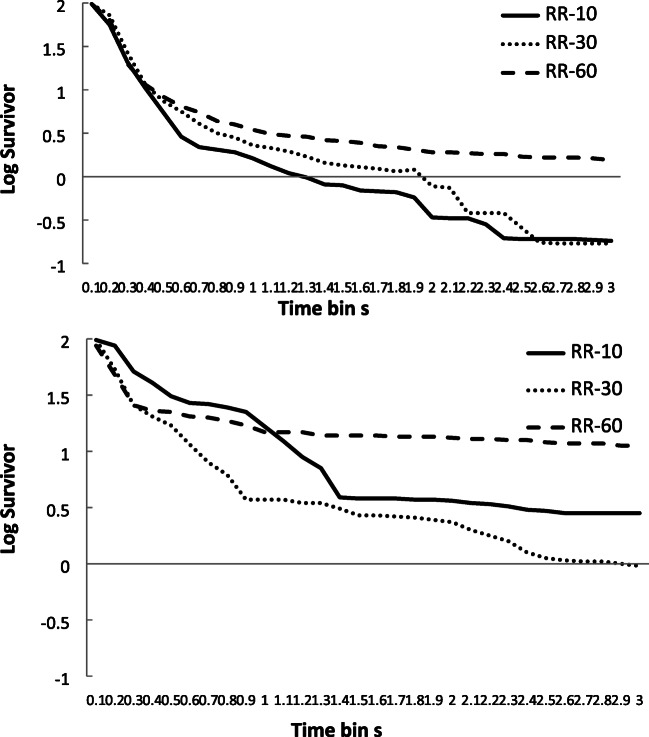
Experiment 1. Mean log survivor plots for percentage of IRTs surviving for the three schedules across successive 100-ms time periods. Top panel = control group (lacking concurrent task. Bottom panel = concurrent group (with concurrent task)

The mean log survivor plots (logs of the percentage of IRTs surviving) for the three schedules are shown in Fig. 2 for the two groups. Inspection of these data reveals a reasonable visual fit to a ‘broken stick’ appearance for the sample. The mean percentage variance accounted for (VAC) by this model was calculated for each participant, for each schedule, and the mean for the sample for the RR-10 schedule was 41.25 (±25.74, range: 2–85); RR-30 = 36.27 (±29.03, range: 2–97); and RR-60 = 37.25 (±30.56, range: 1–84). These mean figures revealed a moderate fit for the sample as a whole, with little difference in goodness of fit between the schedules, although with a large individual variation in the goodness of fit between individuals. In fact, some of the fits for individuals displayed very small levels of variance accounted for by this model. The manners in which these individuals diverged from the pattern predicted by the model tended to be idiosyncratic, although some displayed very shallow flat lines, some displayed very steep flat lines, and some displayed lines with a number of points of inflection. None of these patterns could easily be interpreted as showing a ‘broken stick’ appearance. However, numbers of participants displaying each pattern were too small to draw any firm conclusions about their nature or controlling variables. It should also be noted that the mean survivor plots presented in Fig. 2, show a smoother appearance than is present in many of the individual plots.

**Fig. 3:**
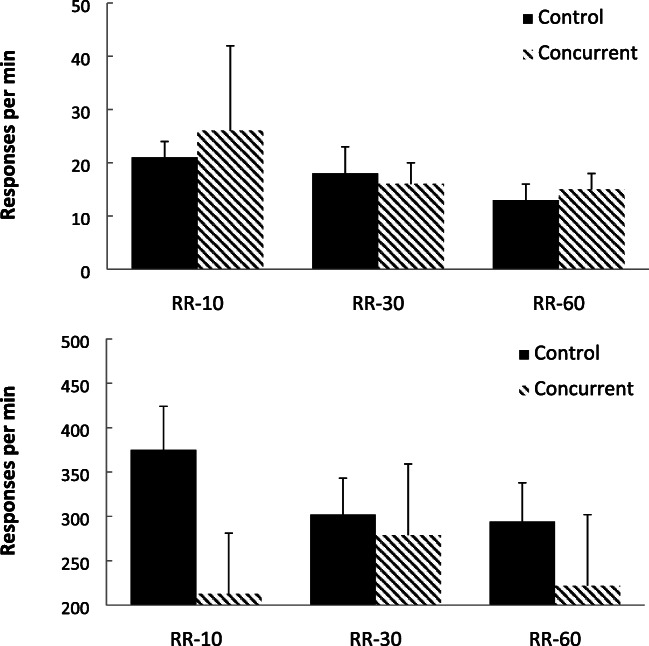
Experiment 1. Group-mean rates of initiation responding (top panel) and within-bout (bottom panel) responding for the three schedules for the two group (Control = no concurrent task using the survivor plot method; Concurrent = counting backwards concurrent task). Error bars = 95% confidence intervals

Figure 3 shows the group-mean rates of bout-initiation (top panel) and within-bout (bottom panel) responding produced by fitting the double exponential equation to the IRTs produced during the 5-min period studied. Inspection of rates of bout-initiation show that both groups demonstrated a reduction in rate as a product of increasing ratio value. The within-bout responses showed a different pattern between the groups, with decreasing rates for the control group lacking a concurrent load, but there was an inverted-U relationship between ratio value and within-bout response rates for the concurrent group.

A two-factor mixed-model ANOVA (Group × Schedule) conducted on the bout-initiation rates revealed a significant main effect of schedule, *F*(2, 76) = 3.74, *p* = .028, η_p_^2^ = .090, [.000, .211], *p(H*_*1*_*/D)* = .555, but no main effect of group, *F* < 1, η_p_^2^ = .0171, [.000, .142], *p(H*_*o*_*/D)* = .837, or interaction between the two factors, *F* < 1, η_p_^2^ = .016, [.000, .090], *p(H*_*o*_*/D)* = .966. Polynomial contrast analyses revealed a significant linear, *F*(1, 38) = 5.07, *p* = .030, η_p_^2^ = .118, [.000, .312], *p(H*_*1*_*/D)* = .561, but not quadratic, *F* < 1, η_p_^2^ = .014, [.000, .153] *p(H*_*o*_*/D)* = .795, trend.

A two-factor mixed-model ANOVA (Group × Schedule) conducted on the within-bout rates revealed no main effect of schedule, *F*(2, 76) = 1.62, *p* = .204, η_p_^2^ = .041, [.000, .139], *p(H*_*o*_*/D)* = .945, but a significant main effect of group, *F*(1, 38) = 6.05, *p* = .019, η_p_^2^ = .137, [.003, .334], *p(H*_*1*_*/D)* = .752, and a significant interaction, *F*(2, 76) = 5.07, *p* = .009, η_p_^2^ = .118, [.009, .246], *p(H*_*1*_*/D)* = .765. Polynomial contrast analyses conducted for the control group revealed a significant linear, *F*(1, 19) = 10.64, *p* = .004, η_p_^2^ = .359, [.047, .585], *p(H*_*1*_*/D)* = .950, but not quadratic, *F*(1, 19) = 2.05, *p* = .169, η_p_^2^ = .097, [.000, .389], *p(H*_*o*_*/D)* = .617, trend. There was no significant linear trend, *F* < 1, η_p_^2^ = .003, [.000, .250], *p(H*_*o*_*/D)* = .813, but a significant quadratic trend, *F*(1, 19) = 4.53, *p* = .047, η_p_^2^ = .192, [.000, .454], *p(H*_*1*_*/D)* = .655, for the concurrent task group.

**Fig. 4:**
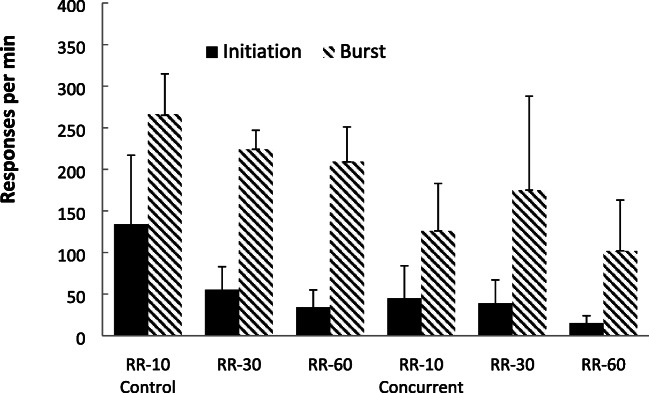
Experiment 1. Group-mean rates of initiation responding and within-bout responding for the three schedules for the two group (Control = no concurrent task; Concurrent = counting backwards concurrent task) using the cutoff method. Error bars = 95% confidence intervals

Figure 4 shows the group-mean rates of bout-initiation and within-bout responding produced by using a cutoff criteria of a 1-s pause from responding. Responses with an IRT of less than 1 s were categorized as within-bout responses, and those following an IRT of 1s or more were classified as bout-initiation responses (Mellgren & Elsmore, 1991; Reed, 2011). Studies have found little impact of the cutoff value selected over a range including 1 s (Reed, 2015a). The rates for each participant were determined by dividing the number of IRTs in each class by the total time taken to emit those responses in that class. For the control group (lacking the concurrent task), within-bout responding decreased as a product of increasing ratio value, but there was little impact of ratio value on rates of bout-initiation responding. For the concurrent group, rates of bout-initiations were highest in the shorter RR schedules and lowest for the RR-60 schedule. In contrast, there was an inverted-U relationship between ratio value and within-bout response rates.

A two-factor mixed-model ANOVA (Group × Schedule) conducted on the bout-initiation rates revealed a significant main effect of schedule, *F*(2, 76) = 11.09, *p* < .001, η_p_^2^ = .226, [.074, .363], *p(H*_*1*_*/D)* = .876, no main effect of group, *F*(1, 19) = 3.84, *p* = .068, η_p_^2^ = .168, [.000, .431], *p(H*_*0*_*/D)* = .675, and no significant interaction between the two factors, *F*(2, 76) = 3.04, *p* = .054, η_p_^2^ = .100, [.003, .225] *p(H*_*0*_*/D)* = .597. Polynomial contrast analyses revealed significant linear, *F*(1, 19) = 8.80, *p* = .010, η_p_^2^ = .317, [.026, .554], *p(H*_*1*_*/D)* = .805, but not quadratic, *F*(1, 19) = 3.17, *p* = .097, η_p_^2^ = .143, [.000, .407], *p(H*_*0*_*/D)* = .712, trends.

A two-factor mixed-model ANOVA (group x schedule) conducted on the within-bout rates revealed significant main effects of group, *F*(1, 19) = 6.82, .*p* = .017, η_p_^2^ = .264[.008:.514], *p(H*_*1*_*/D)* = .823, and schedule, *F*(2, 76) = 3.76, *p* = .028, η_p_^2^ = .173[.000:.211], *p(H*_*1*_*/D)* = .712, and a significant interaction between the two factors, *F*(2, 76) = 3.35, *p* = .040, η_p_^2^ = .160[.000:.119], *p(H*_*1*_*/D)* = .703. Polynomial contrast analyses conducted for the control group revealed significant linear, *F*(1, 19) = 4.43, *p* = .049, η_p_^2^ = .189[.000, .451], *p(H*_*1*_*/D)* = .623, but not quadratic, *F*(1, 19) = 1.17, *p* = .293, η_p_^2^ = .058[.000, .308], *p(H*_*0*_*/D)* = .899, trends. There was no significant linear trend, *F*(1, 19) = 2.18, *p* = .156, η_p_^2^ = .103[.000, .365], *p(H*_*0*_*/D)* = .814, but a significant quadratic trend, *F*(1, 19) = 4.87, *p* = .039, η_p_^2^ = .163[.000, .427], *p(H*_*1*_*/D)* = .734, for the concurrent task group.

These data show differences between the microstructure of human performance on free-operant RR schedules with and without a concurrent load, which were similar to one another when using both forms of analysis. With a concurrent load, performance was similar to that seen in nonhumans, in that bout-initiation rates decreased with increasing ratio values, but within-bout rates had an inverted-U function relationship to ratio values (Brackney et al., 2011; Killeen et al., 2002; Reed, 2011; Sibley et al., 1990). This pattern has also been noted for humans (see Reed, 2015a). However, without a concurrent load, rates of bout-initiation and within-bout responding tended to follow the same pattern as one another, and both were inversely related to the size of the ratio schedule. This might be predicted on the basis of more cognitively demanding processes such as probability matching or response–reinforcer feedback functions (Allan, 1980; Chatlosh et al., 1986; Pérez et al., 2016), but it is not always seen in nonhuman responding on ratio schedules with this range of ratio values (Ferster & Skinner, 1957; Reed & Hall, 1988).

These results suggest a stronger role for the response–reinforcer relationship, such as probability matching, when there was no concurrent task than when there was a concurrent task. The concurrent load also affected within-bout responding to a greater extent than it did bout-initiations. This might suggest that the former type of operant responding may be goal directed to a greater extent than bout-initiation, and so more impacted by factors affecting explicit processing mechanisms.

## Experiment 2

Experiment 2 extended the investigation of concurrent tasks on human free-operant performance to random interval (RI) schedules. On these schedules, overall rates of response typically follow overall rates of reinforcement (Catania & Reynolds, 1968; Herrnstein, 1970; Reed et al., 2018). However, the case for humans without a concurrent task is less clear (Bradshaw et al., 2015; Hayes et al., 1986; Randell et al., 2009). Thus, while a clear relationship between decreasing overall response rates and increasing interval values, similar to that seen in nonhumans, is expected for humans with a concurrent load (Catania & Reynolds, 1968), it is harder to predict the nature of this relationship when there is no concurrent task. In part, this may be because there is a less clear relationship between variations in rates of responding and rates of reinforcement on such schedules (McDowell & Wixted, 1988;), making this explicit/molar process of limited utility in guiding responding even with available resources (Pérez et al., 2016).

For nonhumans (Brackney et al., 2011; Shull, 2011), and humans with a concurrent task (Reed et al., 2018), bout-initiation rates on RI schedules are clearly related to the rate of reinforcement. As this form of responding is hypothesized to reflect relatively mechanistic S–R conditioning, related to the value of the context, then the presence or absence of a concurrent task should not affect this pattern of responding in humans.

Previous studies have demonstrated a less clear relationship between rates of reinforcement and within-bout response rates (Reed, 2011; Shull, 2011). If these responses are thought of as goal directed and under explicit control, then this may be because there is a less clear relationship between variations in rates of responding and rates of reinforcement on such schedules (McDowell & Wixted, 1988), making this explicit/molar process of limited utility in guiding responding. It should be noted that there is some evidence to suggest that longer interval schedules might be expected to drive down rates of responding by reinforcing longer IRTs (Morse, 1966; Reed, 2011), producing this effect in the absence of explicit processing of response–reinforcer relationships.

In summary, the above considerations suggest that with a concurrent task, overall response rates should decrease as the interval value increases, but this should not be so clear in the absence of a concurrent task. Moreover, this pattern should be seen for bout-initiations with or without a concurrent load (given their assumed mechanistic nature), but within-bout responding may decrease more clearly with increasing interval values for the concurrent load group (given their assumed explicit goal-directed nature).

### Method

#### Participants and apparatus

Forty-three participants (24 females and 19 male), with a mean age of 22.10 (±4.34, range: 18–29) years, were recruited as described in Experiment 1. Seven participants were excluded on the basis of having high depression or schizotypy scores, leaving 36 participants in the study. The apparatus was as described in Experiment 1.

#### Procedure

Participants were tested as described in Experiment 1. After presentation of the instructions, each participant was exposed to three schedule types (RI-30s, RI-60s, and RI-120s). Every second of a schedule had the same probability of being the one selected to satisfy the interval requirement, and make reinforcement available for the next response. The probability varied according to the schedule (RI-30 = 1/30; RI-60 = 1/60; RI-120 = 1/120). Each schedule was presented once to each participant for 10 min, with a 30-s intercomponent interval. Each schedule was signaled by the presence of a different colored rectangle. The particular colors used to signal the schedules, and the order of schedule presentation, was randomized. Each response subtracted one point from the ‘points’ box displayed on the screen, and reinforcement consisted of the addition of 60 points to the ‘points’ box. Half of the participants (*n* = 10) were assigned to the concurrent group and had to perform the counting backwards task (Andersson et al., 2002). The other half (*n* = 10) were assigned to the control group, and did not have to complete a concurrent task. Participants completed the questionnaires following completion of the experimental task.

### Results and discussion

**Fig. 5:**
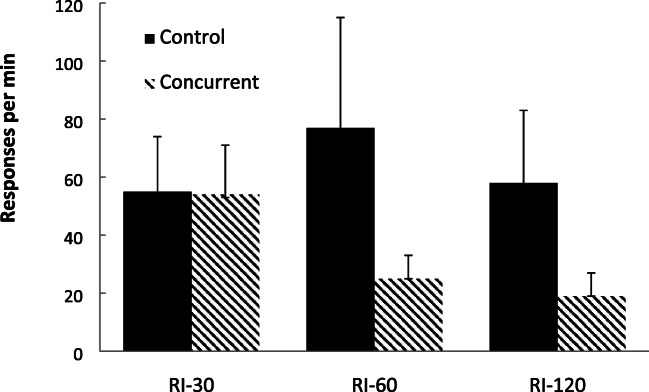
Experiment 2. Group-mean response rates for the three schedules. Control = no concurrent task. Concurrent = concurrent counting backwards task. Error bars = 95% confidence intervals

Figure 5 displays the group-mean rates of responding across the three schedule types, for the two groups, over the last 5-min exposure to each schedule. Inspection of these data for the control group (lacking a concurrent task) reveal that response rates did not reduce systematically as the interval increased. In contrast, for the concurrent group, the response rates did decrease as the interval value increased. A two-factor mixed-model ANOVA (Group × Schedule) revealed a significant main effect of group, *F*(1, 34) = 8.71 *p* = .006, η_p_^2^ = .204, [.020, .411], *p(H*_*1*_*/D)* = .910, no significant main effect of schedule, *F*(2, 68) = 1.62, *p* = .205, η_p_^2^ = .045, [.000, .153], *p(H*_*0*_*/D)* = .940, but a significant interaction, *F*(2, 68) = 3.91, *p* = .025, η_p_^2^ = .103, [.001, .235], *p(H*_*1*_*/D)* = .735. Polynomial contrast analyses revealed no significant linear, *F* < 1, η_p_^2^ = .005, [.000, .160], *p(H*_*0*_*/D)* = .802, or quadratic, *F*(1, 17) = 1.18, *p* = .293, η_p_^2^ = .065, [.000, .331], *p(H*_*0*_*/D)* = .699, trends for the control group. However, there was a significant linear trend, *F*(1, 17) = 23.38, *p* < .001, η_p_^2^ = .579, [.211, .739], *p(H*_*1*_*/D)* = .998, but no significant quadratic trend, *F*(1, 17) = 3.17, *p* = .094, η_p_^2^ = .157, [.000, .432], *p(H*_*0*_*/D)* = .521, for the concurrent group.

These data corroborate previous findings showing that, when a concurrent task is employed, human response rates on RI schedules follow the patterns typically observed for nonhuman participants (Reed et al., 2018)—that is, they decrease as the interval value increases (Catania & Reynolds, 1968; Herrnstein, 1970). In contrast, this effect was not seen in the absence of a concurrent task, which has also been observed in several studies of human RI performance (Lowe, 1979). This effect may be related to the presence of idiosyncratic verbal rules formed by the participants (Bradshaw & Reed, 2012; Hayes et al., 1986; Leander et al., 1968). However, this effect is also predicted by the suggestion that, as there is no strong relationship between the rate of responding and rate of reinforcement on such schedules (McDowell & Wixted, 1988), and this factor may be responsible for human schedule performance when explicit processing is possible (Pérez et al., 2016), this would tend to produce relatively undifferentiated rates of responding.

**Fig. 6:**
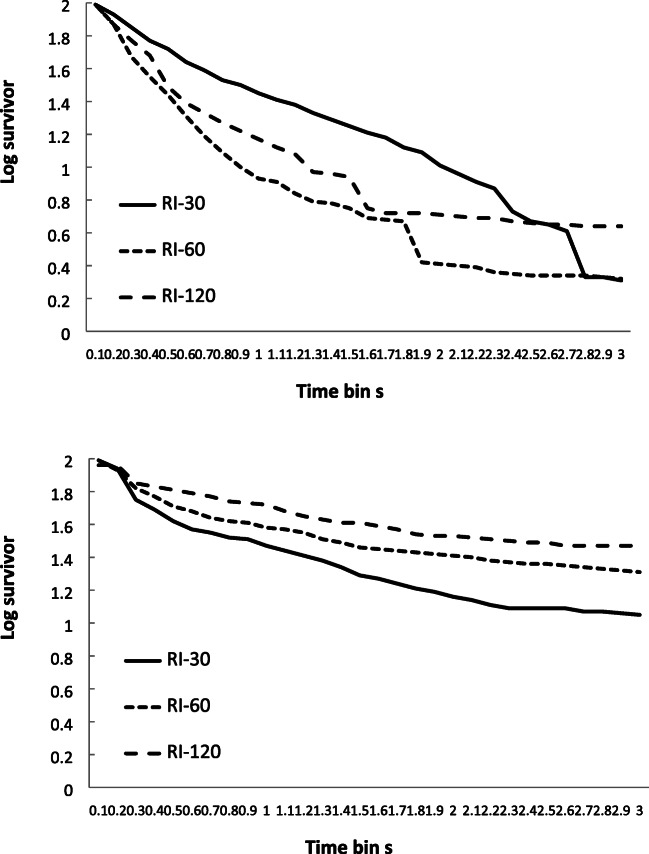
Experiment 2. Mean log survivor plots for percentage of IRTs surviving for the three schedules across successive 100-ms time periods. Top panel = control group (lacking concurrent task. Bottom panel = concurrent group (with concurrent task)

The mean log survivor plots (logs of the percentage of IRTs surviving) for the three schedules are shown in Fig. 6 for the two groups. Inspection of these data reveals an approximation to a visual fit to a ‘broken stick’ appearance for the concurrent group, but this was not so apparent for the control group. The mean VAC for this model was calculated for each participant, for each schedule, and the mean VAC for the RI-30 was 51.39 (±33.02, range: 2–94); RI-60 = 46.33 (±31.36, range: 4–97); and RI-120 = 47.61 (±34.89, range: 1–92). As with Experiment 1, overall there was a moderate fit to the equation, but with a high degree of variability. Again, as with Experiment 1, some of the fits for individuals displayed very small levels of variance accounted for, and there was considerable variation in the manners in which individuals diverged from predicted patterns. Also, the mean survivor plots presented in Fig. 6 show a smoother appearance than is present in many of the individual plots.

**Fig. 7:**
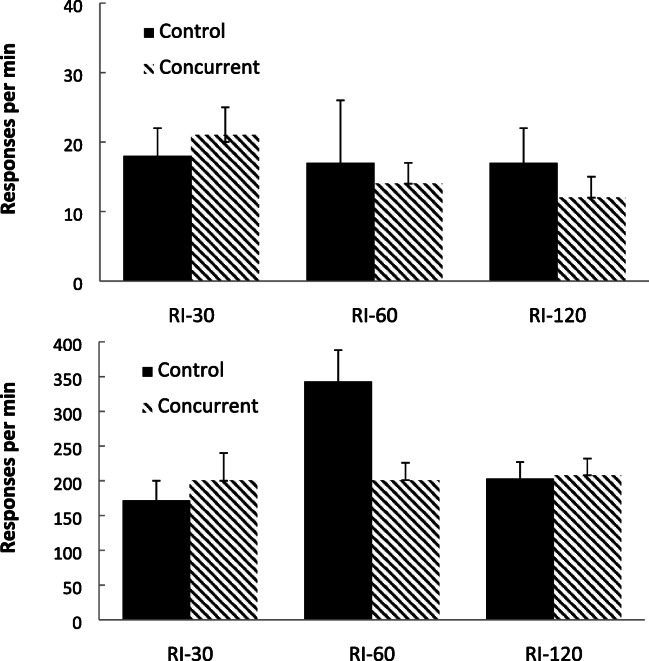
Experiment 2. Group-mean rates of initiation responding (top panel) and within-bout responding (bottom panel) for the three schedules for the two group (Control = no concurrent task using the survivor plot method; Concurrent = counting backwards concurrent task). Error bars = 95% confidence intervals

Figure 7 shows group-mean rates of bout-initiation (top panel) and within-bout (bottom panel) responding, based on the double exponential equation fitting method (Killeen et al., 2002) described in Experiment 1. Bout-initiation rates decreased as the interval increased for both groups, but the case for the impact of the interval value on within-bout rates was less clear, with a slight within-bout response rate decrease noted for the group with the concurrent task.

A two-factor mixed-model ANOVA (Group × Schedule) conducted on the bout-initiation rates revealed a significant main effect of schedule, *F*(2, 68) = 4.00, *p* = .023, η_p_^2^ = .105, [.008, .237], *p(H*_*1*_*/D)* = .829, but no main effect of group, *F* < 1, η_p_^2^ = .014, [.000, .163], *p(H*_*0*_*/D)* = .823, and no significant interaction between the factors, *F*(2, 68) = 2.40, *p* = .093, η_p_^2^ = .061, [.000, .119], *p(H*_*0*_*/D)* = .654. Polynomial contrasts revealed a significant linear, *F*(1, 34) = 36.78, *p* < .001, η_p_^2^ = .684, [.351, .804], *p(H*_*1*_*/D)* = .999, but not quadratic, *F*(1, 34) = 2.719, η_p_^2^ = .138, [.000, .413], *p(H*_*0*_*/D)* = .531, trend.

A two-factor mixed-model ANOVA (Group × Schedule) conducted on the within-bout rates revealed no significant main effect of group, *F* < 1, η_p_^2^ = .001, [.000, .047], *p(H*_*0*_*/D)* = .854, but a marginally significant main effect of schedule, *F*(2, 68) = 2.98, *p* = .058, η_p_^2^ = .080, [.000, .205], *p(H*_*1*_*/D)* = .431, and a significant interaction, *F*(2, 68) = 3.98, *p* = .033, η_p_^2^ = .180, [.000, .345], *p(H*_*1*_*/D)* = .631. Polynomial contrasts conducted for the control group revealed no significant linear, *F*(1, 17) = 2.07, *p* = .168, η_p_^2^ = .205, [.000, .383], *p(H*_*0*_*/D)* = .601, but a significant quadratic trend, *F*(1, 17) = 5.75, *p* = .028, η_p_^2^ = .253, [.000, .514], *p(H*_*1*_*/D)* = .764, trends. In contrast, there was a marginally significant linear, *F*(1, 17) = 3.87, *p* = .065, η_p_^2^ = .074, [.000, .334], *p(H*_*1*_*/D)* = .489, but no quadratic, *F* < 1, η_p_^2^ = .002, [.000, .284], *p(H*_*0*_*/D)* = .806, trend for the concurrent group.

**Fig. 8:**
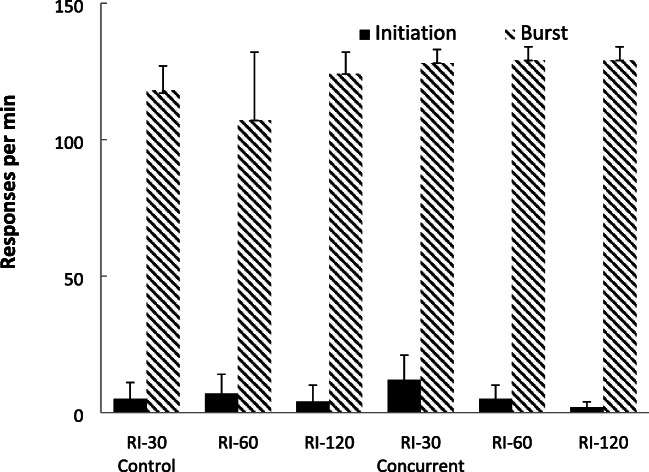
Experiment 2. Group-mean rates of initiation responding and within-bout responding for the three schedules for the two group (Control = no concurrent task; Concurrent = counting backwards concurrent task) using the cutoff method. Error bars = 95% confidence intervals

Figure 8 shows group-mean rates of bout-initiation and within-bout responding, based on using a criterion of a 1-s pause from responding described in Experiment 1. For the concurrent task group, bout-initiations decreased as the interval value grew longer, but there was little impact on within-bout rates. There were few noticeable differences related to schedule value for the control group (lacking a concurrent task).

A two-factor mixed-model ANOVA (Group × Schedule) conducted on the bout-initiation rates revealed a significant main effect of schedule, *F*(2, 68) = 3.80, *p* = .028, η_p_^2^ = .106, [.000, .242], *p(H*_*1*_*/D)* = .839, no main effect of group, *F*(1, 34) = 2.15, *p* = .153, η_p_^2^ = .054, [.000, .229], *p(H*_*0*_*/D)* = .899, but a significant interaction between the factors, *F*(2, 68) = 3.65, *p* = .031, η_p_^2^ = .102, [.000, .238], *p(H*_*1*_*/D)* = .803. Polynomial contrasts conducted for the control group revealed no significant linear, *F* < 1, η_p_^2^ = .029, [.000, .262], *p(H*_*0*_*/D)* = .999, or quadratic, *F* < 1, η_p_^2^ = .012, [.000, .228], *p(H*_*0*_*/D)* = .999 trends. In contrast, there was a significant linear, *F*(1, 17) = 13.38, *p* < .001, η_p_^2^ = .440, [.083, .649], *p(H*_*1*_*/D)* = .999, but not quadratic, *F* < 1, η_p_^2^ = .017, [.000, .246], *p(H*_*0*_*/D)* = .899, trend for the concurrent group.

A two-factor mixed-model ANOVA (Group × Schedule) conducted on the within-bout rates revealed no significant main effects of group, *F*(1, 34) = 1.74, *p* = .196, η_p_^2^ = .044, [.000, .213], *p(H*_*0*_*/D)* = .903, or schedule, *F* < 1, η_p_^2^ = .002, [.000, .097], *p(H*_*0*_*/D)* = .999, but a significant interaction, *F*(2 ,68) = 9.67, *p* < .001, η_p_^2^ = .232, [.062, .379], *p(H*_*1*_*/D)* = .999. Polynomial contrast analyses conducted for the control group revealed a significant linear, *F*(1, 17) = 12.26, *p* < .001, η_p_^2^ = .419, [.068, .634], *p(H*_*1*_*/D)* = .999, but not quadratic, *F*(1, 17) = 2.75, *p* = .116, η_p_^2^ = .132, [.000, .415], *p(H*_*0*_*/D)* = .805, trend. There was no significant linear, *F*(1, 17) = 2.71, *p* > .118, η_p_^2^ = .113, [.000, .388], *p(H*_*0*_*/D)* = .778, or quadratic, *F*(1, 17) = 2.76, *p* = .115, η_p_^2^ = .139, [.000, .416], *p(H*_*0*_*/D)* = .798, for the concurrent group.

Taken together, these data indicate that with a concurrent load, the microstructure of human performance on an interval schedule was similar to that typically noted for rats (Killeen et al., 2002; Shull et al., 2001), increasing interval values resulting in lower rates of bout-initiation, and this factor only marginally affected within-bout rates in a similar manner (Reed et al., 2018). This pattern of results suggests that rate of reinforcement is controlling responding in the expected manner (Herrnstein, 1970; Shull, 2011) for the group with a concurrent load, and may imply that relatively mechanistic processes are operating that are not affected by the reduction in available cognitive resources—such as S–R driving bout-initiations, and IRT reinforcement driving within-bout responding. In the absence of a concurrent load, whereas bout-initiation rates followed rates of reinforcement, within-bout rates did not, suggesting the former but not the latter may be under the control of more explicitly driven processes.

However, while consistent with the view that concurrent loads reduce the likelihood of explicit processes occurring in free-operant schedules, the pattern of these relationships is not as pronounced as it was in Experiment 1, making strong interpretations difficult. In fact, this is predicted by findings reported by Pérez et al. (2016), who suggest that the stronger association between response and reinforcement rates may predispose attention being paid to probabilities of reinforcement on ratio but not interval schedules (see also Baum, 1993).

## Experiment 3

The final experiment examined the impact of a concurrent load task on human responding on RR and RI schedules matched for their rates of reinforcement. A higher rate of responding to RR compared with RI schedules has long been established in nonhumans (Ferster & Skinner, 1957; Peele et al., 1984; Reed, 2001; Zuriff, 1970), and can be noted in human responding under appropriate conditions (see Matthews et al., 1977; Raia et al., 2000; Reed, 2015a). However, in the absence of a concurrent load, or other measures such as a response cost, or control for aberrant personality types, the effect is more elusive for humans (Bradshaw & Reed, 2012; Hayes et al., 1986; Randell et al., 2009). The direct impact of a concurrent load has not been examined on this schedule performance, and the current study aimed to address this issue. Moreover, the microstructure of human yoked RR and RI schedule performance has not been studied under these conditions.

On the basis of previous investigations of RR and RI responding in nonhumans (e.g., Peele et al., 1984; Zuriff, 1970), coupled with the current studies, it might be expected that, with a concurrent load, overall rates and within-bout rates would be higher on the RR than the RI schedule, but that bout-initiation rates would be similar on the two schedules as their rates of reinforcement are matched (Reed, 2015a; Tanno, 2016). In contrast, the effects on overall and within-bout rates might not be apparent in a group lacking a concurrent load. If anything, rates on an RI schedule might be higher as these would be associated with a stronger probability of an outcome given a response given the matched rates of reinforcement and lower overall rates of response (Reed, 1993, 2001).

### Method

#### Participants and apparatus

Forty-four participants (26 female and 18 male), with a mean age of 19.62 (±3.24, range: 18–31) years, were recruited as described in Experiment 1. Four participants were excluded on the basis of having high depression or schizotypy scores, leaving 40 participants in the study. The apparatus and concurrent task were as described in Experiment 1.

#### Procedure

Participants were tested as described in Experiment 1. Following presentation of the instructions, participants were exposed to the experimental task. This comprised eight 2-min exposures to two alternating schedules (an RR-30 and a yoked RI schedule). The RR schedule trial was always presented immediately prior to the yoked RI schedule trial. There were four presentations of the yoked RR-RI pairs. The length of time elapsed between delivery of each reinforcer in an RR trial was stored, and this value became the interval required for reinforcement in the following RI schedule. The procedure of yoking RI trials to preceding RR trials ensured that reinforcement in the RI schedule was delivered after a similar elapse of time that it had taken for the corresponding reinforcer to be awarded on the RR trial. Half of the participants were assigned to the control group, and these were the only contingencies in operation. The other participants were assigned to the concurrent task group, and had to perform the counting backwards task as they were performing the schedule task. After task completion, participants completed the questionnaires.

### Results and discussion

**Fig. 9:**
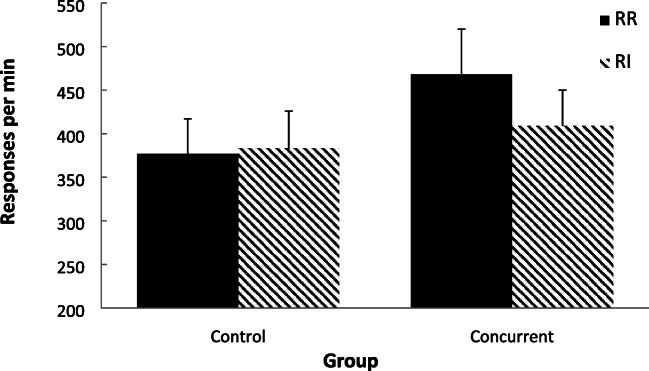
Experiment 3. Group-mean response rates for the two schedules: RR = random ratio; RI = random interval. Control = no concurrent task. Concurrent = concurrent counting backwards task. Error bars = 95% confidence intervals

Figure 9 displays the group-mean rates of responding across the two schedule types, for the two groups, over the last block of exposure to each schedule. Inspection of these data for the control group (lacking any concurrent task) reveals that response rates were not greatly different across the RR and RI schedules. In contrast, the RR schedule had a higher rate of response than the RI schedule for the concurrent group. A two-factor mixed-model ANOVA (Group × Schedule) was conducted on these data and revealed no significant main effect of group, *F* < 1, η_p_^2^ = .001, [.000, .018], *p(H*_*0*_*/D)* = .862, but a significant main effect of schedule, *F*(1, 38) = 4.91, *p* = .033, η_p_^2^ = 114, [.000, .308], *p(H*_*1*_*/D)* = .642, and a significant interaction between the factors, *F*(1, 38) = 7.41, *p* = .010, η_p_^2^ = .163, [.010, .362], *p(H*_*1*_*/D)* = .848. Simple effect analyses conducted between the schedules for each group revealed no significant difference between the schedules for the control group, *F* < 1, η_p_^2^ = .001, [.000, .004], *p(H*_*0*_*/D)* = .852, but a significantly higher rate on the RR schedule than on the RI schedule for the concurrent group, *F*(1, 38) = 12.18, *p* < .001, η_p_^2^ = .247, [.044, .438], *p(H*_*1*_*/D)* = .970.

In order to check that the yoking procedure operated correctly, the mean (standard deviation) for the reinforcement rates for both schedules, on the last block of training, were calculated: RR = 17.88 (±2.55) and RI = 17.77 (±2.59), there was no significant difference between these scores, *t* < 1.

**Fig. 10:**
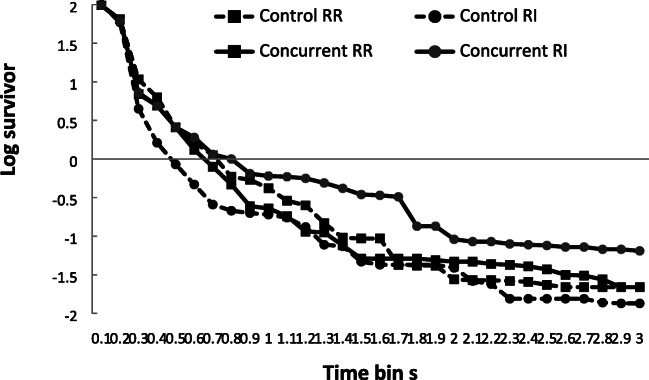
Experiment 3. Mean log survivor plots for percentage of IRTs surviving for the three schedules across successive 100ms time periods

The mean log survivor plots (logs of the percentage of IRTs surviving) for the three schedules are shown in Fig. 10 for the two groups. Inspection of these data reveals a reasonable visual fit to a ‘broken stick’ appearance for the groups as a whole. The mean VAC for this model was calculated for each participant, for each schedule, and the mean VAC for the RR schedule was 30.07 (±18.87, range: 2–80), and for the yoked RI schedule this was 27.60 (±17.77, range: 1–64). These fits were only moderate, and with a large variation between individuals. As for both Experiments 1 and 2, some individual fits displayed small levels of variance accounted for, with there being variation in patterns of divergence from predicted patterns. The mean survivor plots presented in Fig. 10, show a smoother appearance than is present in many of the individual plots.

**Fig. 11:**
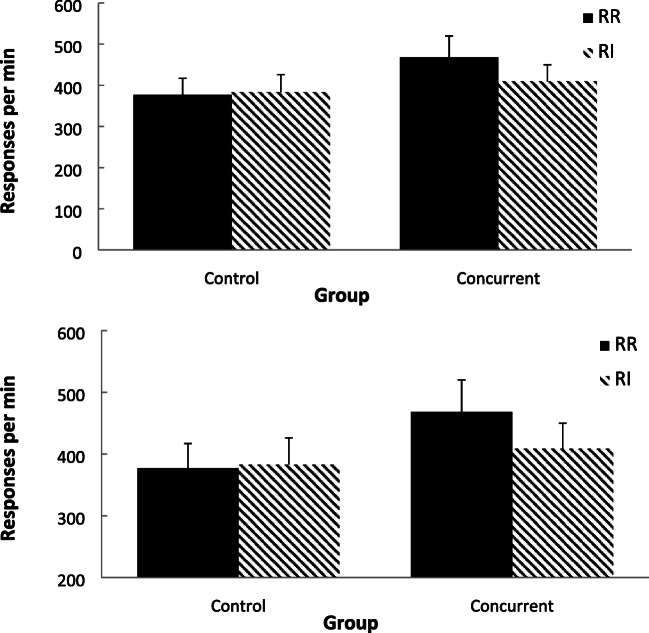
Experiment 3. Group-mean rates of initiation responding (top panel) and within-bout responding (bottom panel) for the two schedules for the two groups (Control = no concurrent task; Concurrent = counting backwards concurrent task) using the survivor plot method. Error bars = 95% confidence intervals

Figure 11 shows group-mean rates of response for the bout-initiation (top panel) and within-bout (bottom panel) responding based on the exponential equation method (Killeen et al., 2002). For the bout-initiation rates, rates of bout-initiations were similar across the two schedules, but within-bout rates were higher for the RR schedule than for the RI schedule for the concurrent group.

A two-factor mixed-model ANOVA (Group × Schedule) conducted on the bout-initiation rates revealed no significant main effects of group, *F*(1, 38) = 1.16, *p* = .287, η_p_^2^ = .030, [.000, .188], *p(H*_*0*_*/D)* = .776, or schedule, *F*(1, 38) = 2.37, *p* = .132, η_p_^2^ = .059, [.000, .237], *p(H*_*1*_*/D)* = .656, or interaction, *F* < 1, η_p_^2^ = .009, [.000, .137], *p(H*_*1*_*/D)* = .842.

A two-factor mixed-model ANOVA (Group × Schedule) conducted on the within-bout rates revealed a significant main effect of group, *F*(1, 38) = 4.99, *p* = .031, η_p_^2^ = .116, [.000, .298], *p(H*_*1*_*/D)* = .651, no main effect of schedule, *F*(1, 38) = 2.88, *p* = .098, η_p_^2^ = .070, [.000, .252], *p(H*_*0*_*/D)* = .594, but a significant interaction, *F*(1, 38) = 4.59, *p* = .039, η_p_^2^ = .108, [.000, .301], *p(H*_*1*_*/D)* = .607. Simple effect analyses of the schedules for each group, revealed no significant difference between the schedules for the control group, *F* < 1, η_p_^2^ = 024, [.000, .088], *p(H*_*0*_*/D)* = .809, but a significantly higher RR rate for the concurrent group, *F*(1, 38) = 7.36, *p* = .005, η_p_^2^ = .162, [.000, .361], *p(H*_*1*_*/D)* = .999.

**Fig. 12:**
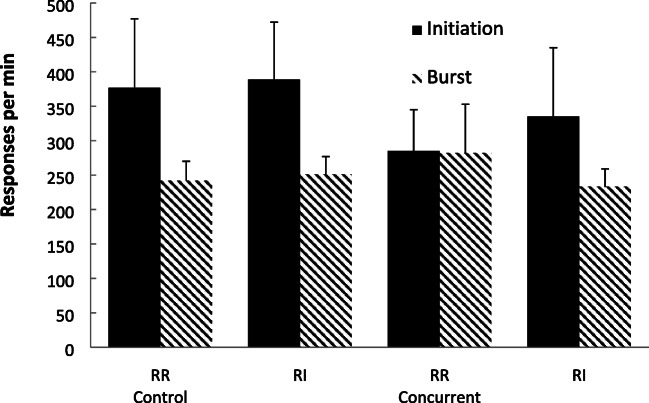
Experiment 3. Group-mean rates of initiation responding and within-bout responding for the two schedules for the two groups (Control = no concurrent task; Concurrent = counting backwards concurrent task) using the cutoff method. Error bars = 95% confidence intervals

Figure 12 shows group-mean rates of response for the bout-initiation and within-bout responding based on the cutoff criteria described in Experiment 1. For the concurrent group, rates of bout-initiations were similar across the two schedules (slightly higher in the RI schedule), but within-bout rates were higher for the RR schedule. There was little difference between the rates of either bout-initiation or within-bout responding for the control group lacking the cognitive load.

A two-factor mixed-model ANOVA (Group × Schedule) conducted on the bout-initiation rates revealed no significant main effects of group, *F* < 1, η_p_^2^ = .054, [.000, .329], *p(H*_*0*_*/D)* = .999, or schedule, *F* < 1, η_p_^2^ = .026, [.000, .283], *p(H*_*0*_*/D)* = .999, or interaction, *F* < 1, η_p_^2^ = .010, [.000, .259], *p(H*_*0*_*/D)* = .999.

A two-factor mixed-model ANOVA (Group × Schedule) conducted on the within-bout rates revealed no significant main effects of group, *F* < 1, η_p_^2^ = .008, [.000, .113], *p(H*_*0*_*/D)* = .999, or schedule, *F*(1, 38) = 1.81, *p* = .186, η_p_^2^ = .046, [.000, .216], *p(H*_*0*_*/D)* = .812, but there was a significant interaction between the two factors, *F*(1, 38) = 3.88, *p* = .049, η_p_^2^ = .091, [.000, .281], *p(H*_*1*_*/D)* = .643. Simple effect analyses of the schedules for each group, revealed no significant difference between the schedules for the control group, *F* < 1, η_p_^2^ = 010, [.000, .142], *p(H*_*0*_*/D)* = .999, but a significantly higher RR rate for the concurrent group, *F*(1, 38) = 5.31, *p* = .027, η_p_^2^ = .127, [.000, .318], *p(H*_*1*_*/D)* = .864.

Taken together, these data from the concurrent group replicate the often-found RR versus RI response rate difference with matched rates of reinforcement for nonhumans (Ferster & Skinner, 1957; Peele et al., 1984; Zuriff, 1970). This difference has also found with humans with a concurrent load (Reed et al., 2018). Moreover, participants with a concurrent load also show higher within-bout, but not bout-initiation, response rates on an RR schedule compared to an RI schedule that has the same rate of reinforcement. This effect has been noted for rats (Reed, 2011, 2015a; Shull, 2011). That bout-initiation rates did not differ between the two schedules is attributed to their sensitivity to rates of reinforcement which did not differ across the schedules (Bowers et al., 2008; Reed, 2011). The lack of difference in the group responding without a concurrent task is similar to the lack of difference noted in previous studies using humans that have not employed any concurrent load (Bradshaw & Reed, 2012; Randell et al., 2009). One feature of the current microanalysis data, that deserves some comment, is that the bout-initiation rates were higher than the within-bout rates when employing the cutoff method (with a potentially similar, although less pronounced, tendency visible when using the survivor method). The reasons why this occurred for this experiment are unclear, other than it employed a different procedure in terms of schedule exposure then the previous studies. Overall, these results suggest, as do those from the previous studies here, that different processes may be engaged in the presence and absence of a concurrent task.

## General discussion

The current series of experiments examined the impact of a concurrent task on human schedule performance, and investigated whether a concurrent task would differentially impact different components of human free-operant responding (Brackney et al., 2011; Mellgren & Elsmore, 1991; Shull, 2011). This investigation was triggered by the lack of data on the impact of this manipulation (Reed, 2015a), and also in the light of the mixed pattern of results that has emerged from the study of human schedule performance (cf. Bradshaw & Reed, 2012; Catania et al., 1982; Hayes et al., 1986; Matthews et al., 1977; Randell et al., 2009). Theoretically, it has been speculated that a concurrent load task takes cognitive resources, and makes explicit processes, involving the tracking of response–reinforcer relationships over time, which may require some resources to keep track of aggregated numbers of responses and reinforcers (Pérez et al., 2016), less likely to control performance (Mitchell et al., 2009; Newell et al., 2007). This would mean that relatively mechanistic processes might operate under conditions of a cognitive load, and make human performance similar to that of nonhumans.

The present data with respect to overall response rates revealed that when a concurrent task was present, human performance on the schedules resembled that seen in nonhumans. Rates of response were an inverted-U function of ratio value (Experiment 1; Ferster & Skinner, 1957; Reed, 2011), were inversely related to interval value (Experiment 2; Herrnstein, 1970; Shull, 2011), and were higher on RR compared to RI schedules (Experiment 3; Peele et al., 1984; Reed, 2001; Zuriff, 1970). In addition, the current studies also noted that bout-initiation responding was related directly to the rate of reinforcement, irrespective of the schedule, and irrespective of the presence of a concurrent load. In contrast, within-bout responding was not so strongly related to this aspect of the schedule. Similar findings are widely found with nonhumans (especially rats) using a variety of analytic procedures (Mellgren & Elsmore, 1991; Pear & Rector, 1979; Shull, 2011; Sibley et al., 1990), and have also been found with humans (Reed, 2015a; Reed et al., 2018). Taken together, these findings suggest that human schedule performance can resemble that of nonhumans, both at a gross and microstructure level, when appropriate procedural steps are taken (see also Raia et al., 2000).

The presence of a concurrent load has been taken to be one of these steps (Reed, 2015a; Reed et al., 2018). In the absence of a concurrent load, overall human schedule performance, and within-bout responding, was not similar to that seen in nonhumans, which has also been noted in several other studies that were conducted without a concurrent task load (Bradshaw et al., 2015; Randell et al., 2009). It has been thought that such a concurrent load might act to suppress the ability to formulate verbal rules about the schedule (Reed, 2015a), which, in turn, interferes with the development of schedule control (Catania et al., 1982; Hayes et al., 1986; Lowe, 1979). This may well be true, and the employment of different concurrent tasks that do, or do not, impact on the ability to form verbal rules could be an interesting avenue to explore (if one fraught with other problems such as equating task difficulty). However, under circumstances when the role of language on schedule performance is not minimized, human performance has not been analyzed as following a particular pattern—but has been assumed to be idiosyncratic to the verbal rule formed (Catania et al., 1982; Hayes et al., 1986). This was not necessarily the case in the current set of studies, where, in the absence of a concurrent load, performance was related to factors such as the probability of a reinforcer given a response, or the correlation between response rates and reinforcement rates (Dickinson & Perez, 2018; Pérez et al., 2016; Reber, 1989). The precise determining factor is currently unclear. That this pattern was not seen in bout-initiation responses suggests that these responses may be controlled by relatively mechanistic processes, such as S–R associations, or contextual conditioning, and are not impacted by reductions in cognitive resources. The use of minimal instructions prior to the study in the current experiments might have helped to reduce the impact of verbal rules (Raia et al., 2000), and reveal this pattern of outcomes.

It has been noted previously that under conditions where processing capacity is limited, humans are less able to integrate information across time (Reed, 2011; Reynolds & Reed, 2011). The current concurrent tasks may have been acting to limit cognitive resources and, consequently, the range of factors that could be paid attention to (Mitchell et al., 2009). This might have driven responding to be controlled by more mechanistic actions of the reinforcement. In terms of the within-bout responses, the patterns of data revealed were consistent with the joint operation of simple IRT reinforcement rules (Peele et al., 1984; Tanno & Silberberg, 2012) and ratio strain (Reed, 2015b). The impact of reinforcement rate on bout-initiation is also often thought to be mediated by the reinforcement conditioning the context, which then drives responding through stimulus–response mechanisms (Nevin & Grace, 2000). It may be that none of these mechanisms requires any great degree of computational/cognitive capacity. However, in the absence of a concurrent task (and in the presence of greater processing ability), strategies such as probability matching, which may require the tracking and integration of information over time would be engaged (see Green & Flowers, 2003; Mitchell et al., 2009). Although the above is clearly speculative, it is consistent with the novel findings emerging from the current series of studies regarding the impact of a concurrent load on human schedule performance.

A relatively novel finding is the presence of two types of responding in human free-operant performance, which has not been well-documented previously. As noted in the general introduction, there are a variety of ways in which this phenomenon can be explored. These effects have been shown previously for nonhumans by adopting a cutoff criterion for responses (Mellgren & Elsmore, 1991; Reed, 2015a; Sibley et al., 1990); those responses following an IRT longer than the cutoff are taken to be bout-initiation responses, and those shorter than the cutoff are taken to be part of an ongoing response bout (Mellgren & Elsmore, 1991; Reed, 2011). Additionally, experimental manipulations can be adopted that physically separate bout-initiation and within-bout responses (Pear & Rector, 1979; Reed, 2011). Again, similar findings emerge from these approaches as from the above cutoff approaches (see Reed, 2011, 2015a). These experimental approaches have the advantage of clearly demarking the two types of responses, but suffer from altering the nature of the contingency from those more often used, with, as yet, unknown effects on performance. The current studies employed both a mathematical modelling approach (Killeen et al., 2002), that showed moderate fit to the data, but it should be noted that when reanalyzing the current data using a cutoff approach, no substantive differences in the qualitative pattern of results was noted. This correspondence suggests that there is little intrinsic advantage for one approach compared to another. Further to this debate, it was noted that fits for individuals did displayed divergences from the pattern predicted by the mathematical model (Killeen et al., 2002). Although these were idiosyncratic—some participants displaying shallow flat lines, some displaying very steep flat lines, and some displaying lines with a number of points of inflection—these divergences might give some insight in the factors controlling such performance. However, much larger sample sizes would be needed to achieve this goal.

A secondary aim was to explore whether the bout-initiation responses, being thought to be controlled by the rate at which reinforcement is delivered (Reed, 2011, 2015a; Shull, 2011; Shull et al., 2001), and potentially by contextual conditioning (Reed et al., 2018), may represent more ‘mechanistic S–R responses’. In this case, they may not be impacted by concurrent loads as they require less explicit processing (see Balleine & Dickinson, 1998). In contrast, within-bout responses are not related to this factor to such a degree (Brackney et al., 2011; Killeen et al., 2002; Reed, 2011; Shull et al., 2001). This corresponds to the oft suggested dichotomy between S–R and goal-directed mechanisms driving responding in instrumental learning (e.g., Balleine & Dickinson, 1998; Dolan & Dayan, 2013; Pérez et al., 2016). The evidence regarding this was less pronounced, and only really strong in Experiment 1, where this prediction was borne out. This may be expected, as it has been suggested that such a relationship between responding and probabilities of an outcome given a response would be stronger on this, than on an interval, schedule (Pérez et al., 2016).

There are limitations surrounding the current studies that need to be acknowledged, and which temper the theoretical conclusions. It has been noted that response rate is much less sensitive to reinforcement rate for pigeons than for rats (Shull, 2005). One interpretation of this species difference is that there is a lower level of implicit concurrent reinforcement for pigeons in their experimental chambers than for rats in theirs. Fits to Herrnstein’s equation show much lower values of alternative reinforcement for pigeons than for rats (Shull, 2005). By this interpretation, the response rates of pigeons are insensitive to rate of reinforcement because there is little else for them to do that provides reinforcement. Additionally, it has been much harder to find clear evidence of bouts in the responding by pigeons than in the responding by rats. Smith et al. (2014) suggested that these two pigeon–rat differences might be related, and examined this possibility by arranging explicit concurrent reinforcement with pigeons. With this concurrent reinforcement, the responding by pigeons came to resemble the performance of rats. It might be that the concurrent task with the humans, here, operated mainly by providing an alternative source of reinforcement, an avoidance contingency of getting chastised for not performing the alternative task well.

Many experiments with college students have shown that their free-operant behavior is variable, fairly insensitive to schedule variations (Catania et al., 1982; Hayes et al., 1986), and very sensitive to control by instructions (Matthews et al., 1977; Shimoff et al., 1986). The current studies show that this is could be procedurally related, but the relatively low effect sizes noted in the current studies, also suggest considerable variability in performance that may reflect the existence of different idiosyncratic performances across the subjects. The instructions might be important in this regard: “To receive points, sometimes you might need to press the spacebar quickly and at other times you might need to press slowly” might have biased participants’ toward probability learning. That is, the instructions provided to participants suggested that they needed to keep their responding variable, because the contingency may change. It has been shown that variability in responding may increase schedule-controlled behavior (Neuringer 2002; Reed, 2007), and may decrease control by self-generated rules in humans (Joyce & Chase, 1990). If the instructions were simply to ‘click to earn points’, and participants were exposed to these contingencies until stable performance was obtained, it is not known whether the results would be the same.

In summary, the current report demonstrated that human schedule performance appears to be controlled by different processes, depending on whether or not a concurrent load task is present. In the presence of a concurrent load, human performance resembles that on nonhumans, and is best explained by current views of the impact of various automatic processes on conditioning. However, in the absence of a concurrent load, probability matching appears to play a strong role in this performance. Moreover, the current results also suggest that different aspects of free-operant responding may be more mechanistically controlled and related to habit (response initiation), and some may be more susceptible to explicit goal-directed control (within-bout responding).
